# Remarkable Controversy

**Published:** 1860-12

**Authors:** 


REMARKABLE CONTROVERSY—That which is going on, in the Cincinnati *Lancet and Observer*, between Prof. John H. Tate, of the College of Medicine and Surgery, and B. F. Richardson, M. D. ("Layman"). The closing paragraph of Dr. R.'s article, in the December number, ought to send one of the parties *out of* the profession and *into* the penitentiary according to the truth or falsity of the insinuation contained.

